# Features of Functionalization of the Surface of Alumina Nanofibers by Hydrolysis of Organosilanes on Surface Hydroxyl Groups

**DOI:** 10.3390/polym13244374

**Published:** 2021-12-14

**Authors:** Mikhail M. Simunin, Anton S. Voronin, Yurii V. Fadeev, Yurii L. Mikhlin, Denis A. Lizunov, Alexandr S. Samoilo, Dmitrii Yu. Chirkov, Svetlana Yu. Voronina, Stas V. Khartov

**Affiliations:** 1 Reshetnev Siberian State University of Science and Technology, 660037 Krasnoyarsk, Russia; chirkov@5d-group.su (D.Y.C.); simkina_svetlana@mail.ru (S.Y.V.); 2 Siberian Federal University, 660041 Krasnoyarsk, Russia; a.voronin1988@mail.ru (A.S.V.); daf.hf@list.ru (Y.V.F.); x_lab@rambler.ru (A.S.S.); 3 Federal Research Center, “Krasnoyarsk Science Center” SB RAS, 660036 Krasnoyarsk, Russia; gipsynanotech@gmail.com (D.A.L.); khartov@5d-group.su (S.V.K.); 4 Bauman Moscow State Technical University, 105005 Moscow, Russia; 5 Institute of Chemistry and Chemical Technology, “Krasnoyarsk Science Center” SB RAS, 660036 Krasnoyarsk, Russia; yumikh@icct.ru

**Keywords:** alumina nanofibers, silanization, tuning interface, polymer nanocomposites, surface chemistry

## Abstract

Small additions of nanofiber materials make it possible to change the properties of polymers. However, the uniformity of the additive distribution and the strength of its bond with the polymer matrix are determined by the surface of the nanofibers. Silanes, in particular, allow you to customize the surface for better interaction with the matrix. The aim of our work is to study an approach to silanization of nanofibers of aluminum oxide to obtain a perfect interface between the additive and the matrix. The presence of target silanes on the surface of nanofibers was shown by XPS methods. The presence of functional groups on the surface of nanofibers was also shown by the methods of simultaneous thermal analysis, and the stoichiometry of functional groups with respect to the initial hydroxyl groups was studied. The number of functional groups precipitated from silanes is close to the number of the initial hydroxyl groups, which indicates a high uniformity of the coating in the proposed method of silanization. The presented technology for silanizing alumina nanofibers is an important approach to the subsequent use of this additive in various polymer matrices.

## 1. Introduction

Nanomaterials are characterized by the fact that their properties are determined not so much by their internal structure as by the structure of their surface [1]. It is clear that thermodynamic potentials are determined by the nature of the transition to an equilibrium state and the interaction of nanoparticles with the external environment. Thus, by controlling the structure of the nanoparticles’ surface, one can significantly change the nature of their interaction with the external environment. The addition of filamentary nanoparticles leads to hardening of the compounded matrices [2]. This makes it possible to use such additives in polymeric materials for the tuning of their strength [3,4,5,6,7,8,9].

During the compounding of polymers with nanomaterials, the latter are introduced into the polymer matrix and distributed over it. Depending on the nanoparticle’s surface properties, it can both bind and order polymer chains around itself [10] and, on the contrary, can loosen the structure of the polymer, creating an additional free volume [11]. To achieve the first property, the nanomaterial’s surface should ideally be as similar as possible to the polymer’s structure, so as to bind the polymer matrix to the surface of the nanomaterial by covalent bonds. In the second case, the nanomaterial’s surface should be extremely antagonistic to the polymer material, so that the functional groups on its surface repel partially or completely all the elements of the polymer chain links.

Since the middle of the twentieth century, a method of surface modification has been known owing to the hydrolyzable organosilicon compounds’ interaction with a hydrophilic surface [12]. This method is often referred to as finishing or silanizing. In this case, an organosilicon compound is used. In this case, some of the bonds with silicon are hydrolysable—as a rule, these are siloxane bonds with the simplest organic methyl or ethyl radicals. These molecules’ elements serve to bond with the surface through hydrolysis on the surface’s OH–groups and the Si–O-surface bonds’ formation. Another part of the silicon’s valence electrons is associated with the silane’s organic part, which carries on one or more functional groups. It is this organic or functional part that determines the surface’s properties after silanization [13,14,15,16]. Today, the chemical industry has accumulated a sufficient range of different silanes to place a given functional group on a given surface at the required temperature or reaction rate [17].

One of the interesting nanostructured components are alumina nanofibers obtained by the technology of melting aluminum oxidation [18]. Alumina nanofibers make it possible to strengthen polymer materials [19,20]. However, the problem of introducing nanomaterials into a polymer matrix by itself cannot provide an effective solution for the resulting composite materials without matching the nanomaterial and the polymer matrix’s surface states. It is the consistency of the nanomaterial and the polymer matrix surface states that determines the quality of desired properties’ transfer to the resulting polymer composite.

Modern methods of surface functionalization are very diverse and are based on the chemical interaction of a primer with surface groups [21]. In addition to organosilanes, functionalizing agents such as polymers [22], amines [23], fatty acids [24], and the like can be found. The mobility of polymer chains can lead to the formation of a loosened interface between the additive and the polymer matrix. The target group for amines is predominantly acid sites on the surface, and the amphoteric nature of alumina can lead to low efficiency of this method in our case. The use of fatty acids limits the potential use to non-polar matrices only. Thus, the silanization process makes it possible to obtain a wide range of functional groups on the surface of alumina nanofibers.

Depending on the surface functionalization procedure, nanoparticles exhibit completely different properties. During silanization, the process of hydrolysis of silanes on the surface can occur in the presence of an aqueous adsorbate and moist air. This can lead to the appearance of silica aggregates around the nanofibers, which will loosen the interface between the additive and the matrix. It is important to ensure a strong bond between the nanoparticle and the polymer matrix and to prevent any inclusions in the interface. For silanes, this can be realized in the case when the hydrolysis of alkoxide groups occurs strictly on the surface of the nanoparticle. Then, the functional group interacting with the polymer matrix will be directly attached to the surface of the nanofibers. The aim of our work is to study an approach to silanization of nanoparticles in which functional groups bind only to the surface of nanofibers, and hydrolyzed silanes do not create extraneous aggregates on the surface. This article describes the approaches and method for treating alumina nanofibers with silanes in order to control the interaction interface between the polymer and the additive.

## 2. Materials and Methods

### 2.1. Materials

Alumina nanofibers were obtained from ENAW-1199 aluminum using the molten aluminum oxidation technology [18], similar to those we used earlier [19,20], obtaining nanofibers having no more than 1% impurities. The silanization process was carried out with silanes manufactured by Millipore Sigma, Hamburg, Germany; these are 4-aminobutyltriethoxysilane (ABES) with a purity of 98%, vinyltrimethoxysilane (VTMS) with a purity of 98%; and 3-methacryloxypropyltrimethoxysilane (MAMS) with a purity of 98%, and 3-glycidyloxypropyltrimethoxysilane (GlyMS) with a purity of 98% (Evonik, Wesseling, Germany). We also used anhydrous toluene (Sigma-Aldrich Chemie, Steinheim am Albuch, Germany) with a purity of 99.8% as a solvent. ABES was used for precipitation of butylamine functional groups, VTMS for vinyl functional groups, MAMS for methacrylic functional groups, and GlyMS for epoxypropyl functional groups.

### 2.2. Silanization

**Figure 1:**
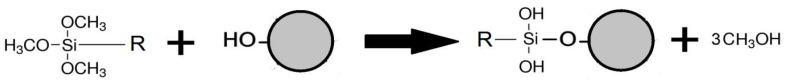
Scheme of binding functional groups onto the surface of alumina nanofibers.

The selection of silanization modes made it possible to develop an optimal procedure for the treatment of alumina nanofibers with silanes. For each silanziation, 10 g of alumina nanofibers was taken and dried at a temperature of 140 °C in a ShS-40-02 SPU drying oven for about 8 h in order to remove all adsorbed water from the surface, and at the same time to preserve hydroxyl groups in the surface structure. Then, the nanofibers were dispersed in toluene using an overhead stirrer based on the SGR-1 YHCHEM reactor with a stirring speed of 1000 rpm within 90 min. Next, the suspension was heated to 80 °C with stirring at 600 rpm within 40 min. The selected silanes were added to the obtaining dispersion in the amount of 5 g. Thus, in the obtained dispersion, the only place where silane can be hydrolyzed is the surface of alumina nanofibers according to the scheme (Figure 1). The silane’s remaining alkoxy groups can either also bind to the surface, owing to hydrolysis in the appropriate microstates’ presence at the interface, or hydrolyze after the end of the silanization process, when the solvent is removed.

Heating is used to accelerate the silane’s diffusion in the medium and the speed of its binding to the surface of nanofibers. After thermal stabilization of the alumina nanofibers’ dispersion in toluene, the required silane is added with stirring. After stirring the mixture for an hour, it is cooled, rinsed with toluene, and decanted. Toluene residues are removed by drying at a temperature of 120–140 °C in a drying oven within 8 h.

### 2.3. Simultaneous Thermal Analysis

Simultaneous thermal analysis was used to determine functional groups on the surface of alumina nanofibers by weight loss and the characteristic heat flow to which this process leads. Samples were analyzed on a NETZCH Jupiter 449 (Selb, Germany). Samples in the form of a powder were placed in a Pt crucible on a differential sensor with an identical Pt crucible as a reference, and then heated in a thermostabilized oven at a rate of 10 °C/min in air flow of 100 sccm. The sensor was also blown with a protective argon flow of 100 sccm.

### 2.4. XPS

X-ray photoelectron spectra were measured with a SPECS instrument equipped with a PHOIBOS 150 MCD 9 hemispherical analyzer at an electron take-off angle of 90° with the pass energy of 10 eV for high-resolution spectra and 20 eV for survey spectra. The pressure in an analytical chamber was in the range of 10^−9^ mBar. The spectra were excited by monochromatic Al Kα irradiation (1486.6 eV) of an X-ray tube. The C 1s, O 1s, Al 2p, and Si 2p spectra were fitted with Gaussian–Lorentzian peak profiles after subtraction of a Shirley-type background utilizing the CasaXPS software (version 2.3.16, Casa Software, Teignmouth, UK).package. The samples were not exposed to preliminary annealing by testing at 200 °C. This could disrupt the functional groups under study.

## 3. Results

### 3.1. Raw Alumina Nanofibers

**Figure 2:**
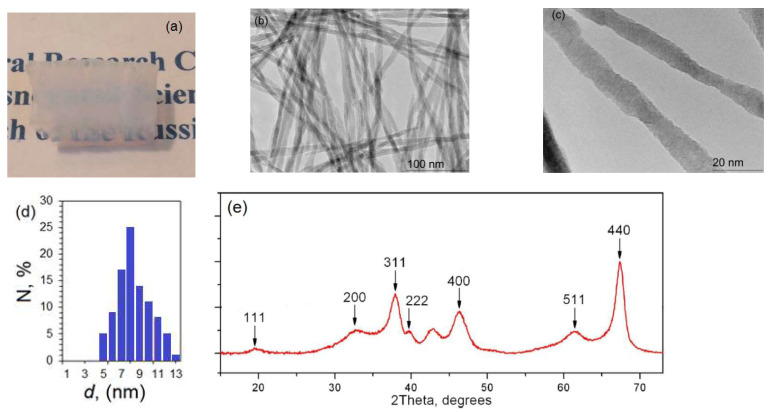
Morphology of the raw alumina nanofibers. (**a**) Photo, (**b**) scanning electron microscopy, (**c**) transmission electron microscopy, (**d**) histogram of a sample of nanofibers by diameter, and (**e**) X-ray phase analysis.

Alumina nanofibers are synthesized from molten aluminum in air humidity of 98%. The obtained nanofibers are similar to those we used earlier [19,20]. Alumina nanofibers are synthesized with a highly oriented texture (Figure 2a–c) and have an extremely high aspect ratio of length in the range of centimeters at nanometer diameters. The nanofiber diameter is determined by the synthesis mode and has a relatively narrow statistical distribution (Figure 2d) in the diameter range from 5 nm to 15 nm. Nanofibers are single crystals or chains of single crystals with a γ-Al_2_O_3_ structure (Figure 2e). This set of peaks is characteristic of γ-Al_2_O_3_ having the stoichiometric formula Al_2.67_O_4_, which belongs to the cubic system like a simple spinel structure with unit cell parameters a = b = c = 7.906 Å. Space group of symmetry Fd3m.

It should be noted briefly that alumina nanofibers retain all the chemical properties characteristic of alumina-gel-oxide compositions, an example of which can be anodic alumina [25] or sedimentation pseudoboehmite [26]. Nanofibers are well wetted by most solvents and form stable dispersions both in water and in alcohols or surfactant solutions.

**Figure 3:**
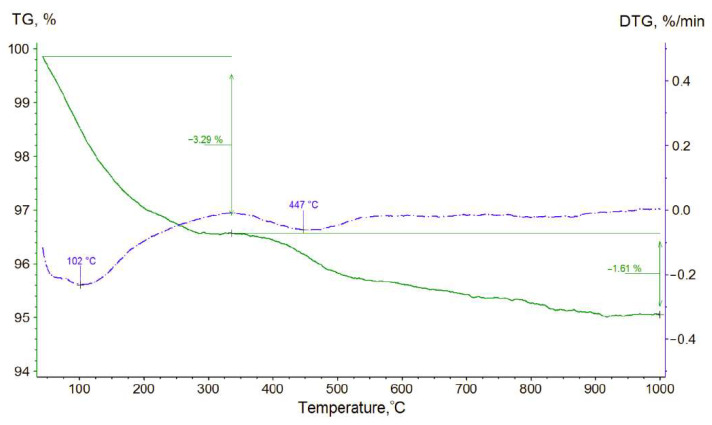
Characteristic graph of simultaneous thermal analysis for raw alumina nanofibers.

Annealing of alumina nanofibers leads to the removal of water adsorbate and destruction of AlOOH on their surface; the higher the annealing temperature, the more chemically stable the material becomes. These data were confirmed by simultaneous thermal analysis (Figure 3).

The loss in mass on the TG and DTG plots, characterized by the minimum of the derivative plot at 102 °C, indicates the loss of water by the material, which, under normal conditions, is physically bound to the surface of nanofibers in the form of an adsorbate. Further mass loss, extended in temperature, is explained by a chemical reaction,

2AlOOH → Al_2_O_3_ + H_2_O (1)

which leads to a loss of the remaining 1.61% of the mass from the sample. Most likely, AlOOH is located only on the nanofibers surface, as loss of mass does not lead to the destruction of the material’s fibrous structure. It should also be noted that a weight loss of ~5% as a result of annealing is generally characteristic of some alumina nanofibers [27].

### 3.2. Fixation of Functional Groups on the Surface of Alumina Nanofibers

The surface’s functional groups of alumina nanofibers were determined by the XPS method (Table 1). In the alumina nanofibers’ samples coated with butyl-amine functional groups, such elements as carbon; oxygen; nitrogen; silicon; and, naturally, aluminum are identified. In the butyl-amine functional group (C_4_H_15_NSi≡), silicon and oxygen are contained in equal amounts, which is in good agreement with the reported data. The proportion of silicon in the sample is 2.5 % and that of nitrogen is 2.3 %; therefore, with some conventions, we can take their ratio as 1:1, i.e., corresponding to the stoichiometry of the functional group. The proportion of silicon in relation to carbon (26.0%) in the sample, that is, the silicon–carbon ratio, is 1:10, which, however, differs from the silicon/carbon ratio in the butyl-amine functional group, which is equal to 1:4. Excess carbon in the samples studied by the XPS method is most often due to adsorbed CO_2_ and other organic pollutants. Usually, they are disposed of by annealing at a temperature of 200–300 °C, but in our case, this would lead to the destruction of functional groups. Finally, the close ratio of the atomic fractions of oxygen (45.2%) and aluminum (23.9%) should be noted, as the bulk of the sample consists of Al_2_O_3_; their ratio in the sample should be 3:2 or 1.5:1, however, it is easy to see in the sample that their ratio is 1.9:1. The excess oxygen in the sample is also associated with the air adsorbate, which is composed of H_2_O and CO_2_.

In the alumina nanofibers’ samples coated with epoxypropyl functional groups, such elements as carbon, oxygen, silicon, and aluminum are identified. In the epoxypropyl functional group (C_6_H_11_O2Si≡) and beyond, we do not have such a bright identifier as nitrogen. The silicon proportion in the sample is 1.4%; the ratio of this element with carbon (29.0%) is 1:21. This excess of carbon relative to the stoichiometric ratio should also be attributed to various carbonaceous contaminants. Oxygen in the sample is more related to the constituent element of alumina nanofibers than by the presence in the functional group of the ratio of the atomic fractions of oxygen (48.8%) and aluminum (20.8%); in the sample, their ratio is 2.35:1 and excess oxygen cannot be attributed in any way to its contribution from the epoxypropyl functional group. The excess oxygen in the sample is also associated with the air adsorbate, which is composed of H_2_O and CO_2_.

In alumina nanofibers’ samples coated with methacrylic functional groups (C_7_H_11_O_2_Si≡), such elements as carbon, oxygen, silicon, and aluminum are identified. The silicon proportion in the sample is 1.5%; the ratio of this element with carbon (32.5%) is 1:22. Here, too, the excess of carbon relative to the stoichiometric ratio should be explained by various carbon-containing adsorbate. Oxygen in the sample is more related to the constituent element of alumina nanofibers than by the availability in the functional group of the ratio of the atomic fractions of oxygen (46.5%) and aluminum (19.5%); in the sample, their ratio is 2.38:1 and excess oxygen is also associated with air adsorbate, which consists predominantly of H_2_O and CO_2_.

Finally, in alumina nanofibers’ samples coated with vinyl functional groups (C_2_H_3_Si≡), we also identify such elements as carbon, oxygen, silicon, and aluminum. The silicon proportion in the sample is 2.7%; the ratio of this element with carbon (20.0%) is 1:7.3. Oxygen in the sample is more related to the constituent element of alumina nanofibers than by the presence in the functional group of the ratio of the atomic fractions of oxygen (51.8%) and aluminum (25.5%); in the sample, their ratio is 2:1. In this sample, we also have a deviation from the stoichiometric proportion between the elements due to the presence of air adsorbate.

In all materials, the silicon peaks observed near 102 eV have such a partial contribution (~2%) that its amount strongly differs from stoichiometry (the number of OH groups is estimated at about 8–14%) and does not correspond to the data of simultaneous thermal analysis. Most likely, there is a significant influence of the background, and because of its contribution, we observe a quantitative discrepancy. Functional groups can be characterized as chemisorbed molecules, and their number, proportional to the specific surface of nanofibers, is comparable to the amount of air adsorbate. Nevertheless, we see a similarity in the values of silicon and nitrogen for the samples of alumina nanofibers functionalized with amino groups, which allows us to conclude that the target functional group is contained on the surface of the nanofibers. Generally, the presence of silicon lines in the spectra indicates that the silanization of alumina nanofibers was successful.

### 3.3. Thermal Properties of Functionalized Alumina Nanofibers

**Figure 4:**
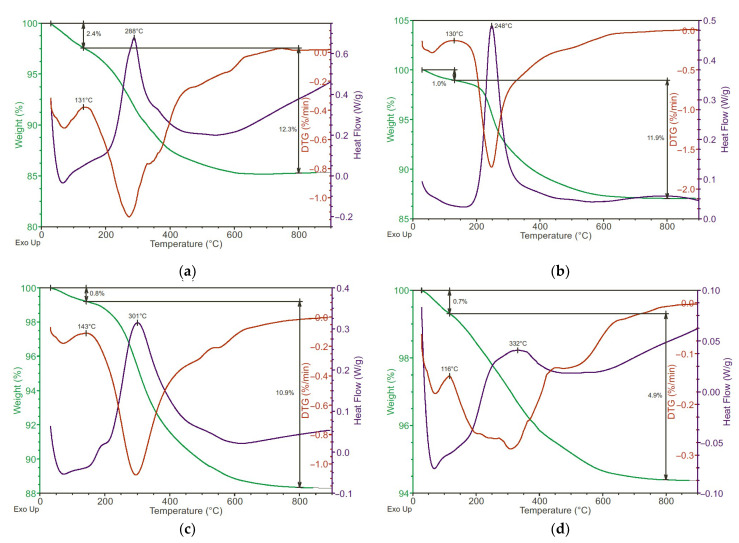
Simultaneous thermal analysis of alumina nanofibers. (**a**) Alumina nanofibers coated by butylamine groups, (**b**) alumina nanofibers coated by epoxypropyl groups, (**c**) alumina nanofibers coated by metacryl groups, and (**d**) alumina nanofibers coated by vinyl groups.

A uniform study of samples by the method of simultaneous thermal analysis will allow a uniform description of all the main processes (Figure 4). When heated in ambient air, the samples lose adsorbed water, which is illustrated by both a peak in the derivative of the weight change and a downward convex peak of the heat flux. However, the water evaporation from the samples smoothly transforms into the combustion of functional groups in ambient conditions. This is illustrated by a peak in the derivative of weight change, and an exothermic peak in heat flux. Oxidation of functional groups and carbon chains ends after 800 °C and the weight of the samples does not change anymore.

The sample with vinyl functional groups has the least amount of water, the evaporation of which ends at about 116 °C. This is because the vinyl functional group is the least polar of the entire sample. The vinyl group’s stability is characterized by an exothermic peak of the heat flux at 332 °C, which is associated with its oxidation. The methacrylic functional group is freed from the adsorbate at a temperature of about 143 °C, and its combustion is characterized by an exothermic peak of the heat flux at 301 °C. The epoxy functional group is freed from the adsorbate at a temperature of about 130 °C, and its combustion is characterized by an exothermic peak of the heat flux at 248 °C. The lowest combustion temperature is due to the instability of the epoxy functional group above 200 °C. Finally, the butylamine functional group is freed from the adsorbate at a temperature of about 131 °C, and its combustion is characterized by an exothermic peak in heat flux at 288 °C. These are the samples that are characterized by the largest proportion of adsorbate; this can be explained by the amine centers’ polarity.

### 3.4. Silanization Stoichiometry

If we assume that a silane is added to each hydroxyl group, then the number of functional groups will be equal to the number of hydroxyl groups on the surface of alumina nanofibers. We obtain the mass fraction of hydroxyl groups *ω_OH_* = 1.61% from the data of raw alumina nanofibers’ thermal analysis. The equality in the amounts of hydroxyl and functional groups leads to the equality of their proportions, which can be expressed through the mass fraction of hydroxyl groups:

(2) $$X=\frac{\omega_{OH}M_{Al2O3}}{M_{OH}\left(1-\omega_{OH}\right)}$$

Here, the letter *M_OH_* is the molecular weight of the hydroxyl group, and *M*_*Al*2*O*3_ is the molecular weight of aluminum oxide. If, during thermal analysis, hydroxyl groups completely evaporate, then during the oxidation of functional groups, silicon remains on the surface of nanofibers and, taking this into account, the mass fraction of the removed part of the functional groups is determined by the ratio below:

(3) $$\omega_{R}=\frac{XM_{R}}{XM_{R}+XM_{Si}+M_{Al2O3}}$$

Here, the letter *M_R_* denotes the molecular weight of the functional group removed in the process of simultaneous thermal analysis, whereas *M_Si_* denotes the molecular weight of silicon.

Data calculated from stoichiometric ratios are summarized in Table 2 for comparison with STA data.

From the presented comparison, it can be seen that treatment with VTMS and GlyMS leads to the formation of functional groups on the surface of alumina nanofibers in an amount close to stoichiometric. There are fewer methacrylic functional groups in the silanized nanofiber sample than is suggested by stoichiometry. This can be explained by the instability of these functional groups. There are much more butylamine functional groups in the sample than is suggested by stoichiometry. The excess of butylamine groups is most likely associated with the aminosilanes’ dimerization [28].

## 4. Conclusions

The interaction of organosilanes with the surface of alumina nanofibers by hydrolysis on hydroxyl groups was shown in this article. The silanes’ selection allows us to state that most of these primers effectively modify the surface, which can be used for their introduction into various polymers, both non-polar using VTMS and polar with various variants of interaction with the polymer matrix.

An interesting fact is the stoichiometric closeness of the number of hydroxyl and functional groups. This indicates that the bond of silanes with the surface of alumina nanofibers occurs during the hydrolysis of only one alkoxysilane group, whereas the rest, apparently, are oxidized after the silanization process. Although it is quite possible that there are microstates in which a silane combines simultaneously with two or three hydroxyl groups, they are still realized much less frequently owing to the electrostatic repulsion of hydroxyl groups from each other. Thus, the silane molecule combines with only one hydroxyl group for purely topological reasons.

It has also been shown that the silanization process is applicable to metal oxide nanomaterials such as alumina nanofibers. An important step in this procedure is the preliminary drying of the nanofibers. As a large specific surface area of the material accumulates a large amount of air adsorbate, it will necessarily lead to the hydrolysis of silanes and the loosened binding of their functional groups to the surface of alumina nanofibers.

Thus, the article presents an efficient technique for silanizing alumina nanofibers with various organosilanes to improve their interface with target polymers. The improvement is achieved by the uniformity of coating the alumina nanofibers with functional groups (Figure S1). The stoichiometric agreement between the number of hydroxyl groups on raw nanofibers and functional groups on silanized nanofibers indicates the perfection of the obtained functionalization. This is groundwork for the use of alumina nanofibers silanized according to the presented technology as an additive in polymers.

## Figures and Tables

**Table 1:** XPS data of raw alumina nanofibers and treated with different silanes.

Sample	C 1s	Al 2p	O 1s	Si 2p	N 1s
	at. %	at. %	at. %	at. %	at. %
ABES -NFA	26.0	23.9	45.2	2.5	2.3
MAMS-NFA	32.5	19.5	46.5	1.5	-
VTMS-NFA	20.0	25.5	51.8	2.7	-
GlyMS-NFA	29.0	20.8	48.8	1.4	-

**Table 2:** Comparison of the functional groups’ stoichiometric amount with that obtained in the samples.

Functional Group Names	Molecular Weight of Silane Primer	Molecular Weight of Linked Group	STA Weight Fraction of Group%	Stoichiometric Weight Fraction of Group%
Hydroxyl		17.01	1.61	
Vinyl	148.23	55.23	4.9	4.92
Methacryl	248.24	155.24	10.9	12.70
Epoxypropyl	236.34	143.34	11.9	11.84
Aminobutyl	235.40	100.40	12.3	8.60

## Supplementary Materials

The following are available online at https://www.mdpi.com/article/10.3390/polym13244374/s1, Figure S1: For TEM, alumina nanofibers were dispersed in 99.99% pure isopropanol by sonication for 5 min, after which the dispersion was applied to a carbon-coated gold grid and dried.
