# Supplementary material for: Features of Functionalization of the Surface of Alumina Nanofibers by Hydrolysis of Organosilanes on Surface Hydroxyl Groups
Source: Polymers (Basel). 2021 Dec 14;13(24):4374. doi: 10.3390/polym13244374 (PMC8707266; doi:10.3390/polym13244374)
Supplement: Supplementary file 1 [file polymers-13-04374-s001.zip › polymers-1465743-supplementary.pdf]

polymers

Supplementary materials

Features of Functionalization of the Surface of Alumina Nanofibers by Hydrolysis of Organosilanes on Surface Hydroxyl Groups

M. M. Simunin [1,2,3*], A. S. Voronin [2,3,4], Yu. V. Fadeev [2,3], Yu. L. Mikhlin [5], D. A. Lizunov [3], A. S. Samoilo [2], D. Yu. Chirkov [1,3], S. Yu. Voronina [1] and S. V. Khartov [3]

1 Reshetnev Siberian State University of Science and Technology, Krasnoyarsk 660037, Russia; chirkov@5d-group.su (D.Y.C.); simkina_svetlana@mail.ru (S.Y.V.)
2 Siberian Federal University, Krasnoyarsk 660041, Russia; a.voronin1988@mail.ru (A.S.V.); daf.hf@list.ru (Y.V.F.); x_lab@rambler.ru (A.S.S.)
3 Federal Research Center, Krasnoyarsk Science Center SB RAS, 660036, Russia; gipsynanotech@gmail.com (D.A.L.); khartov@5d-group.su (S.V.K.)
4 Bauman Moscow State Technical University, 105005 Moscow, Russia
5 Krasnoyarsk Science Center SB RAS, Institute of Chemistry and Chemical Technology, Krasnoyarsk 660036, Russia; yumikh@icct.ru
* Correspondence: michanel@mail.ru; Tel.: +7-913-197-39-26

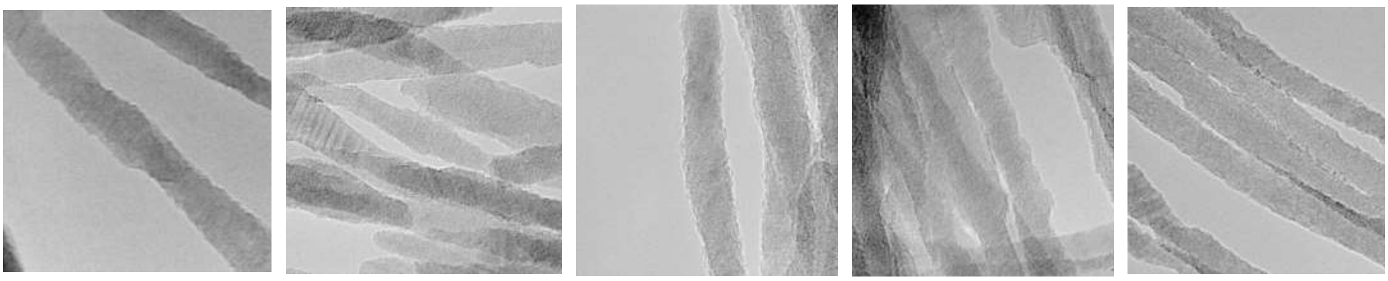

TEM of raw NFA | TEM VTMS-NFA | TEM MAMS-NFA | TEM GlyMS-NFA | TEM ABES-NFA

Figure S1. For TEM, alumina nanofibers were dispersed in 99.99% pure isopropanol by sonication for 5 minutes, after which the dispersion was applied to a carbon-coated gold grid and dried.
